# Multi‐Omic Analysis Reveals the Impact of Bortezomib in Hyperleukocytic Acute Myeloid Leukemia

**DOI:** 10.1002/cam4.70438

**Published:** 2024-11-25

**Authors:** Jinxian Wu, Xinqi Li, Bei Xiong, Wanyue Yin, Ruihang Li, Guopeng Chen, Linlu Ma, Xiqin Tong, Xiaoyan Liu, Fuling Zhou

**Affiliations:** ^1^ Department of Hematology Zhongnan Hospital of Wuhan University Wuhan Hubei China; ^2^ Research Center for Lifespan Health Wuhan University Wuhan Hubei China

**Keywords:** bortezomib, hyperleukocytic acute myeloid leukemia, NF‐kappa B pathway, proteomics

## Abstract

**Background:**

Hyperleukocytic acute myeloid leukemia (HL‐AML) is associated with early complications and high mortality rates, highlighting the urgent need for more effective therapeutic strategies.

**Methods:**

This study conducted label‐free proteomic analysis on serum from HL‐AML and non‐HL AML (NHL‐AML) patients, integrating the data with the OHSU transcriptomic database. Flow cytometry was used to evaluate the in vitro impact of bortezomib. The in vivo effectiveness of bortezomib was assessed using the patient‐derived xenograft (PDX) model of HL‐AML.

**Results:**

Through integrated analysis of serum proteomics and transcriptomics, we observed an abnormal enrichment of the NF‐kappa B pathway in HL‐AML, suggesting its potential as a novel therapeutic target. Given that bortezomib is an inhibitor of the NF‐kappa B pathway, HL‐AML bone marrow cells were treated with varying concentrations of bortezomib (0, 5, 10, and 20 nM) in vitro. The results indicated a significant cytotoxic effect of bortezomib on HL‐AML cells, accompanied by increased apoptosis rates and decreased proliferation. Co‐administration of bortezomib with the frontline clinical chemotherapeutic regimen of daunorubicin and cytarabine (DA regimen) significantly extended mouse survival. Bone marrow immunophenotyping showed reductions in CD45^+^ and CD33^+^ cell populations, indicating disease amelioration. Immunohistochemical analysis further confirmed the inhibitory effect on the NF‐kappa B pathway, as evidenced by reduced levels of P‐IKBα and P‐p65 proteins, validating the proposed therapeutic mechanism.

**Conclusions:**

These data suggest that combination therapy involving bortezomib and the DA regimen may represent a promising strategy for HL‐AML.

## 1 Introduction

Acute myeloid leukemia (AML) constitutes a clonal hematopoietic stem cell malignancy characterized by the accumulation of immature progenitor cells with compromised differentiation, leading to the impairment of hematopoietic function [1, 2]. Approximately 20% of individuals diagnosed with AML exhibit leukocytosis, with peripheral blood leukocyte counts exceeding 50 × 10^9/L or 100 × 10^9/L. This group is referred to as hyperleukocytic AML (HL‐AML) [3, 4]. HL‐AML emerges as an oncological emergency due to its elevated risk of early mortality resulting from leukemic infiltration of organs, leukocyte stasis syndrome, diffuse intravascular coagulopathy, and tumor lysis syndrome. HL‐AML manifests with an abrupt onset, rapid progression, relatively low remission rate, and a notably high early mortality rate, reaching up to 40% within 1 week [5, 6, 7]. It is classified as a high‐risk subtype of acute leukemia. Due to its severe early lethality, certain patients with HL‐AML face challenges in undergoing conventional treatment or novel drug therapy promptly. HL‐AML displays significant heterogeneity in terms of morphology, immunophenotype, cytogenetic and epigenetic features, as well as response to therapy [4, 8, 9]. Therefore, investigating the characteristics and potential therapeutic targets of HL‐AML patients assumes paramount importance.

Serum proteins play dual roles as contributors to diseases and potential therapeutic targets. Proteomic analysis stands as a crucial method for identifying biomarkers in biological and biomedical samples [10, 11, 12]. This technique entails the exhaustive characterization of the proteome of a cell or biofluid, facilitating the identification and subsequent bioinformatics analysis of proteins exhibiting differential expression. Serum‐based proteomics, in particular, offers an advantage in unveiling the progression of diseases. An increasing body of research indicates that combining transcriptomics and proteomics technologies to analyze the complex molecular mechanisms of hematologic malignancies is a promising approach. However, specific serum proteomic analysis of HL‐AML has yet to be reported.

Here, we conducted a label‐free proteomic analysis of serum from patients with HL‐AML and NHL‐AML, integrating the results with publicly available transcriptomic data. The findings unveiled a pronounced enrichment of the NF‐kappa B pathway in HL‐AML. Additionally, bortezomib played a pivotal role in the treatment of HL‐AML patients. This research offers unprecedented insights into the molecular distinctions between HL‐AML and NHL‐AML, deepening our comprehension of their mechanisms at the molecular level and providing guidance for refining therapeutic strategies against this malignancy.

## 2 Materials and Methods

### 2.1 Patient Population

Participants were recruited from Wuhan University Zhongnan Hospital in Wuhan, China, diagnosed and classified as AML based on the 2016 World Health Organization criteria. The study included newly diagnosed AML patients. These patients are stratified into HL‐AML and NHL‐AML categories based on whether their white blood cell (WBC) counts exceed 50 × 10^9/L. Informed consent was obtained, and ethical approval was secured from Zhongnan Hospital of Wuhan University. Detailed patient information is provided in Table S1.

### 2.2 Proteomics Analysis

Label‐free LC–MS/MS quantitative proteomic analysis was conducted by Jingjie PTM BioLab. Bone marrow samples were collected from four specimens from patients with HL‐AML and four specimens from patients with NHL‐AML, followed by centrifugation at 3000 rpm for 10 min. The serum was then stored at −80°C until further use. After thawing at −80°C, the samples were centrifuged at 4°C for 10 min at 12,000 g to remove cellular debris. The supernatant was transferred into fresh centrifuge tubes, and high‐abundance proteins were depleted according to the manufacturer's protocol (Pierce Top 14 Abundant Protein Depletion Spin Columns Kit, Thermo Scientific). Protein concentration was determined using the BCA kit. An aliquot of the protein from each sample was used for enzymatic digestion. Peptides were solubilized in mobile phase A and separated via an EASY‐nLC 1200 ultra‐performance liquid chromatography system (UPLC). Mobile phase A consisted of an aqueous solution containing 0.1% formic acid and 2% acetonitrile, while mobile phase B comprised 0.1% formic acid and 90% acetonitrile. The liquid‐phase gradient settings were: 0–68 min, 4%–20% B; 68–82 min, 20%–32% B; 82–86 min, 32%–80% B; and 86–90 min, 80% B. The flow rate was maintained at 500 nL/min. Peptide fragments were separated via the UPLC system, ionized using the NSI Ion Source, and subsequently analyzed using the Orbitrap Exploris 480 mass spectrometer. Protein sequences were identified via Proteome Discoverer (v2.4.1.15) software, employing Homo_sapiens_9606_PR_20210721.fasta database (78,120 sequences). The false discovery rate (FDR) for protein, peptide, and PSM identification were all set to 1%. Significantly different proteins (*p* < 0.05) underwent subsequent Gene Ontology (GO) and Kyoto Encyclopedia of Genes and Genomes (KEGG) analysis.

### 2.3 Data Collection

Gene expression profiles and clinical parameters were obtained from cBioPortal. Differentially expressed genes (DEGs) were identified using the limma package in R, with criteria of adjusted *p* < 0.05, and |log2FC| > 0.5. Functional annotation utilized GO and KEGG pathway through the “ClusterProfiler” R package.

### 2.4 Cell Counting, Apoptosis, and Cell Proliferation

Cell counting was conducted utilizing trypan blue cell counting. For apoptosis assessment, the Annexin V‐FITC/PI apoptosis assay kit (Lianke Biotechnology) was employed in accordance with the manufacturer's instructions. BrdU staining was performed following the manufacturer's protocol using the BD BrdU Flow Kit.

### 2.5 Animal Experimentation

NSG mice (female, 5 weeks old) were procured from Jiangsu Jicui Biotechnology Co. Ltd. Bone marrow blood from HL‐AML patients was subjected to gradient centrifugation to isolate mononuclear cells, which is instrumental in model construction. Each mouse was intravenously injected with 1 × 10^7 HL‐AML cells to induce leukemia. Successful establishment of a PDX HL‐AML model was confirmed upon the observation of HL‐AML cells in mouse bone marrow smears. Subsequently, the mice were randomly allocated into four groups, with a 1‐week dosing regimen. The DA group received daily intraperitoneal injections of cytarabine (30 mg/kg) for 7 consecutive days. Additionally, on Days 1, 3, and 5, intraperitoneal injections of daunorubicin (1.5 mg/kg) were administered. The bortezomib (1 mg/kg) group received intraperitoneal injections of bortezomib on Days 1, 2, 4, and 6. The DA combined with the bortezomib group underwent 7 consecutive days of intraperitoneal cytarabine administration, daunorubicin injections on Days 1, 3, and 5, and bortezomib injections on Days 1, 2, 4, and 6. The control group received equivalent volumes of physiological saline with matching frequency. At the end of the experiment, mice were executed and tissues were harvested for further study. Information on flow cytometry antibodies is provided in Table S2.

### 2.6 Statistical Analysis

Statistical analyses were performed using GraphPad Prism (version 8.0) or *R* script. In survival experiments, mouse survival rates were analyzed using the log‐rank test. Differences between groups were analyzed using two‐tailed *t*‐tests. Data are presented as mean ± standard deviation (SD).*p < 0.05, **p < 0.01, ***p < 0.001.

## 3 Results

### 3.1 Serum Proteomic Analysis of HL‐AML Patients

**FIGURE 1:**
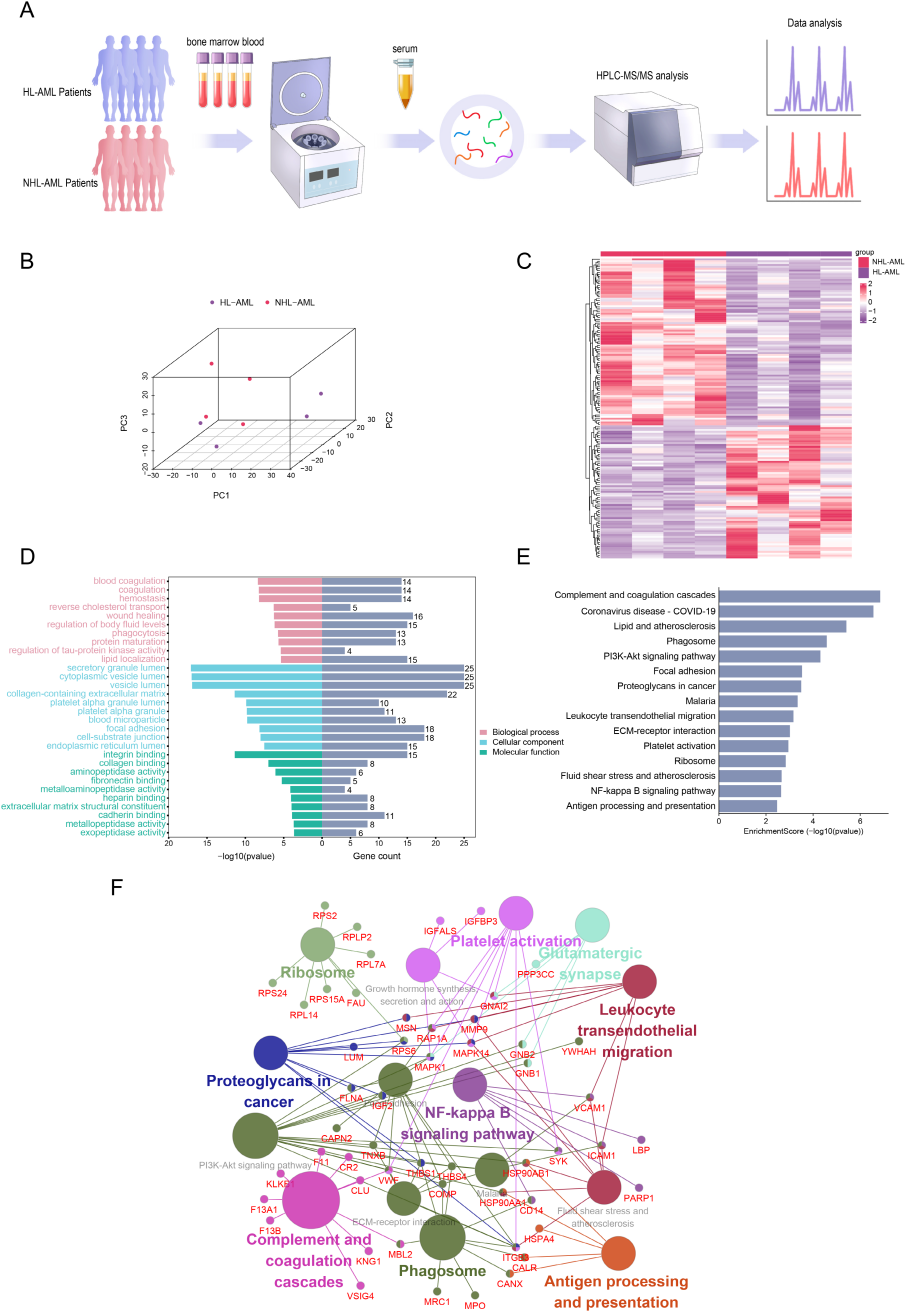
Proteomic analysis of serum from bone marrow in HL‐AMLand NHL‐ AML patients. (A) Schematic representation of general protein quantification through mass spectrometry. HL‐AML, *n* = 4; NHL‐AML, *n* = 4. (B) PCA illustrating the correlation between bone marrow serum proteomic data of HL‐AML and NHL‐AML patients. (C) Heatmap displaying differentially expressed proteins, with a total of 71 upregulated and 89 downregulated proteins identified. (D) GO enrichment analysis of differentially expressed proteins. (E) Bar graphs showing KEGG pathways of differentially expressed proteins. (F) The KEGG pathway enrichment of differentially expressed proteins is visualized with nodes of varying sizes. Nodes with larger sizes denote the KEGG pathway terms, while smaller nodes represent the associated proteins. Nodes with multiple colors indicate involvement in more than one pathway.

AML patients were stratified into high leukocytosis (WBC > 50 × 10^9/L) and non‐high leukocytosis (WBC< 50 × 10^9/L) based on their WBC count. Label‐free proteomic analysis of bone marrow serum samples from four cases of HL‐AML patients and four cases of NHL‐AML patients was conducted (Figure 1A). Following quality control standardization of the dataset, a total of 1297 serum proteins were detected. Principal component analysis (PCA) was initially employed to delineate the relationship between HL‐AML and NHL‐AML; serum proteomics distinctly differentiated HL‐AML samples from NHL‐AML samples, as illustrated in Figure 1B. To elucidate the enriched proteins in the serum of HL‐AML patients, a comparative analysis of the quality‐controlled 1297 serum proteins was undertaken, identifying 160 differentially expressed serum proteins (*p* < 0.05). Among these, 71 proteins were upregulated, while 89 proteins were downregulated (Figure 1C and Table S3). Enrichment analysis was performed to explore the biological functions of these differentially expressed proteins. GO analysis revealed biological processes (BP) encompassing “blood coagulation” and “hemostasis,” while the most abundant terms in cellular components (CC) were “secretory granule lumen” and “cytoplasmic vesicle lumen,” and in molecular functions (MF), it was “integrin binding” (Figure 1D and Table S4). Furthermore, KEGG pathway enrichment analysis indicated significant enrichment in classical tumor‐related pathways such as the PI3K‐Akt signaling pathway, leukocyte transendothelial migration, and NF‐kappa B signaling pathway (*q* < 0.05, Figure 1E). To further elucidate the functions of the differentially expressed proteins, we performed KEGG pathway analysis using ClueGO (version 2.5.7) (https://apps.cytoscape.org/apps/cluego). The ClueGO enrichment analysis indicated that the differentially expressed proteins were primarily involved in the complement and coagulation cascades, NF‐kappa B signaling pathway, and leukocyte transendothelial migration (*p* < 0.05, Figure 1F).

### 3.2 The NF‐Kappa B Pathway as a Candidate Therapeutic Target for HL‐AML

**FIGURE 2:**
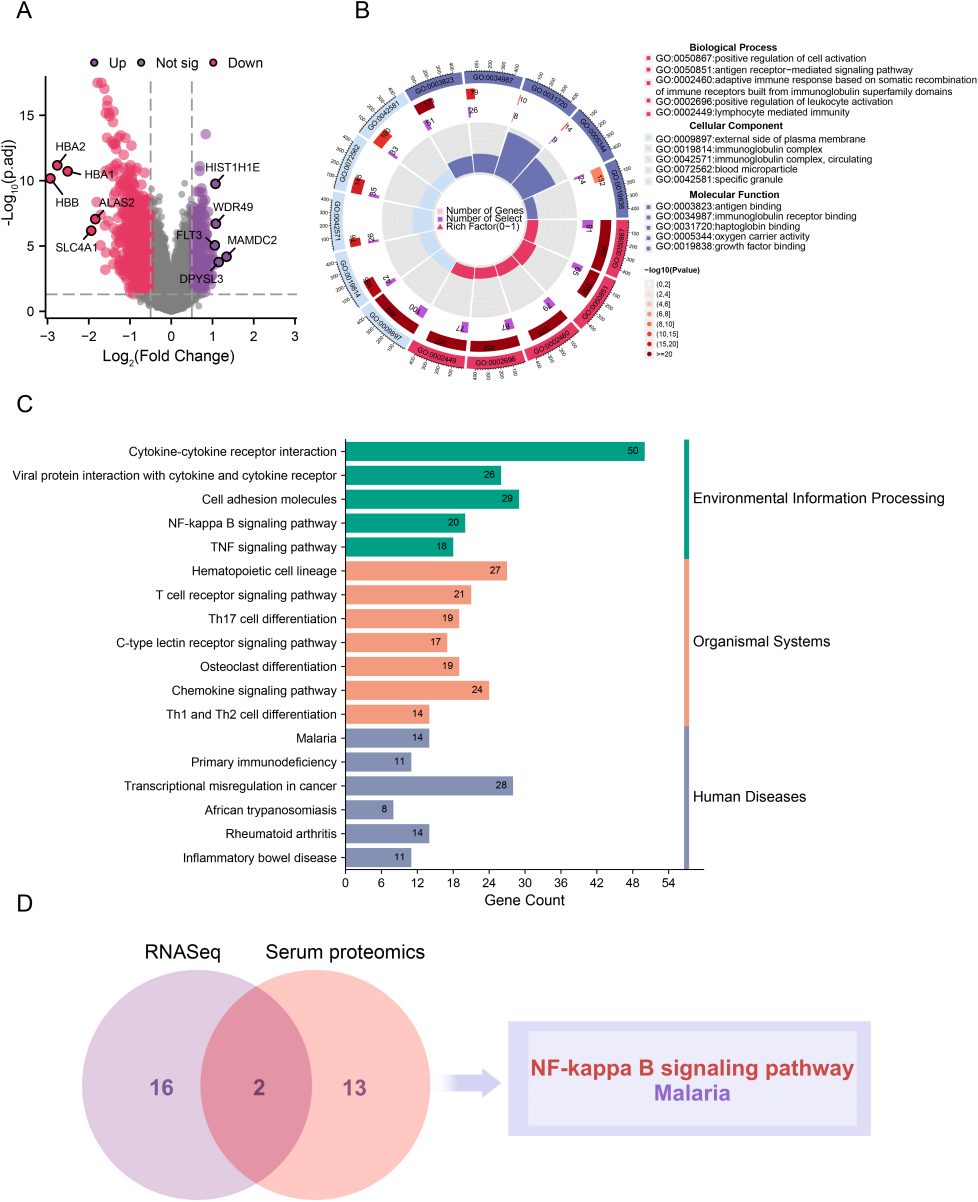
Transcriptomic and proteomic analysis reveals aberrant enrichment of the NF‐kappa B pathway. (A) Volcano plot illustrating differential gene expression between HL‐AML and NH‐AML. (B) GO enrichment analysis based on DEGs. (C) KEGG pathway enrichment analysis based on DEGs. (D) Intersection of enriched pathways between proteomic and transcriptomic analyses.

In the OHSU‐AML cohort, we identified 1211 DEGs (adjusted *p* < 0.05, and |log2FC| > 0.5). Among these, 387 genes were upregulated, and 824 genes were downregulated. The volcano plot is presented in Figure 2A. GO analysis revealed the enrichment of these DEGs in positive regulation of cell activation, external side of the plasma membrane, and antigen binding (Figure 2B and Table S5). KEGG results elucidated potential biological functions, encompassing a total of 18 significantly enriched pathways across environmental information processing, organismal systems, and human diseases (*q* < 0.05, Figure 2C). By intersecting the enriched pathways from the proteomic and transcriptomic analyses, we observed enrichment of the NF‐kappa B pathway at both the gene and protein levels. Given this convergence, we subsequently focus on unraveling the potential significance of the NF‐kappa B pathway in HL‐AML patients (Figure 2D).

### 3.3 Inhibition of HL‐AML Cell Proliferation by Bortezomib In Vitro

**FIGURE 3:**
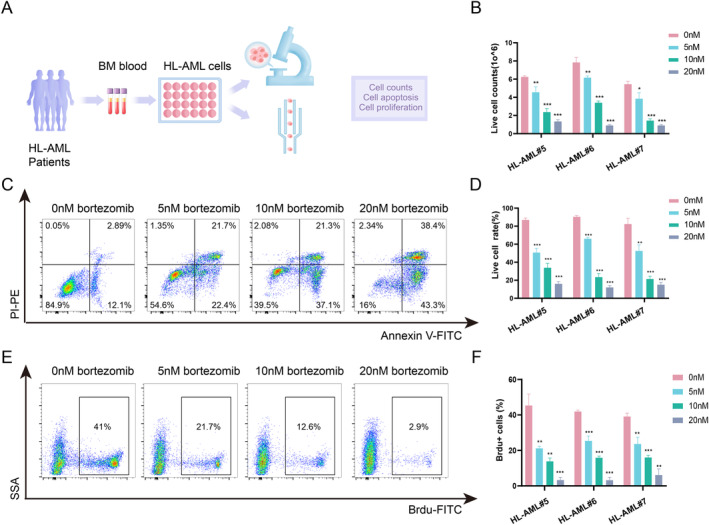
In vitro validation of the cytotoxic effect of bortezomib on HL‐AML cells. (A) Experimental setup for assessing the cytotoxic effect of bortezomib on HL‐AML cells. Bone marrow samples were collected from three HL‐AML patients and subjected to gradient centrifugation to isolate mononuclear cells, which were subsequently used for cellular experiments. (B)Treatment with different concentrations of bortezomib (0, 5, 10, and 20 nM) for 48 h, followed by cell viability assessment using Trypan Blue staining. (C, D) Cells treated with bortezomib for 48 h, stained with Annexin V and PI, and apoptotic rates analyzed by flow cytometry. (E, F) Analysis of cell proliferation through BrdU staining. *p < 0.05, **p < 0.01, ***p < 0.001.

Following the notable enrichment of the NF‐kappa B pathway in HL‐AML, we aimed to assess whether its inhibition could represent an effective therapeutic strategy. Bortezomib, a proteasome inhibitor with well‐known properties of inhibiting the NF‐kappa B pathway, has been approved for the treatment of a variety of hematologic malignancies [13, 14]. To this end, we collected bone marrow cells from three HL‐AML patients and treated HL‐AML cells with increasing concentrations of bortezomib (0–20 nM) to examine the killing effect of bortezomib on HL‐AML cells (Figure 3A). The results showed a significant decrease in viable HL‐AML cells and a notable increase in apoptosis rates (Figure 3B–D). Further, BrdU assays assessing cell proliferation reveal a marked inhibition of HL‐AML cell proliferative capacity by bortezomib (Figure 3E,F). Additionally, we compared the cytotoxic effects of bortezomib on HL‐AML versus NHL‐AML. Bone marrow cells were collected from three patients with HL‐AML and three with NHL‐AML, followed by isolation of mononuclear cells using density gradient centrifugation. After treating the cells with bortezomib for 48 h, flow cytometry was used to assess apoptosis, revealing that bortezomib exerted a stronger cytotoxic effect on HL‐AML compared to NHL‐AML (Figure S1). These results indicate the cytotoxicity of bortezomib on HL‐AML cells.

### 3.4 Bortezomib Combined With DA Regimen Suppresses HL‐AML Growth In Vivo

**FIGURE 4:**
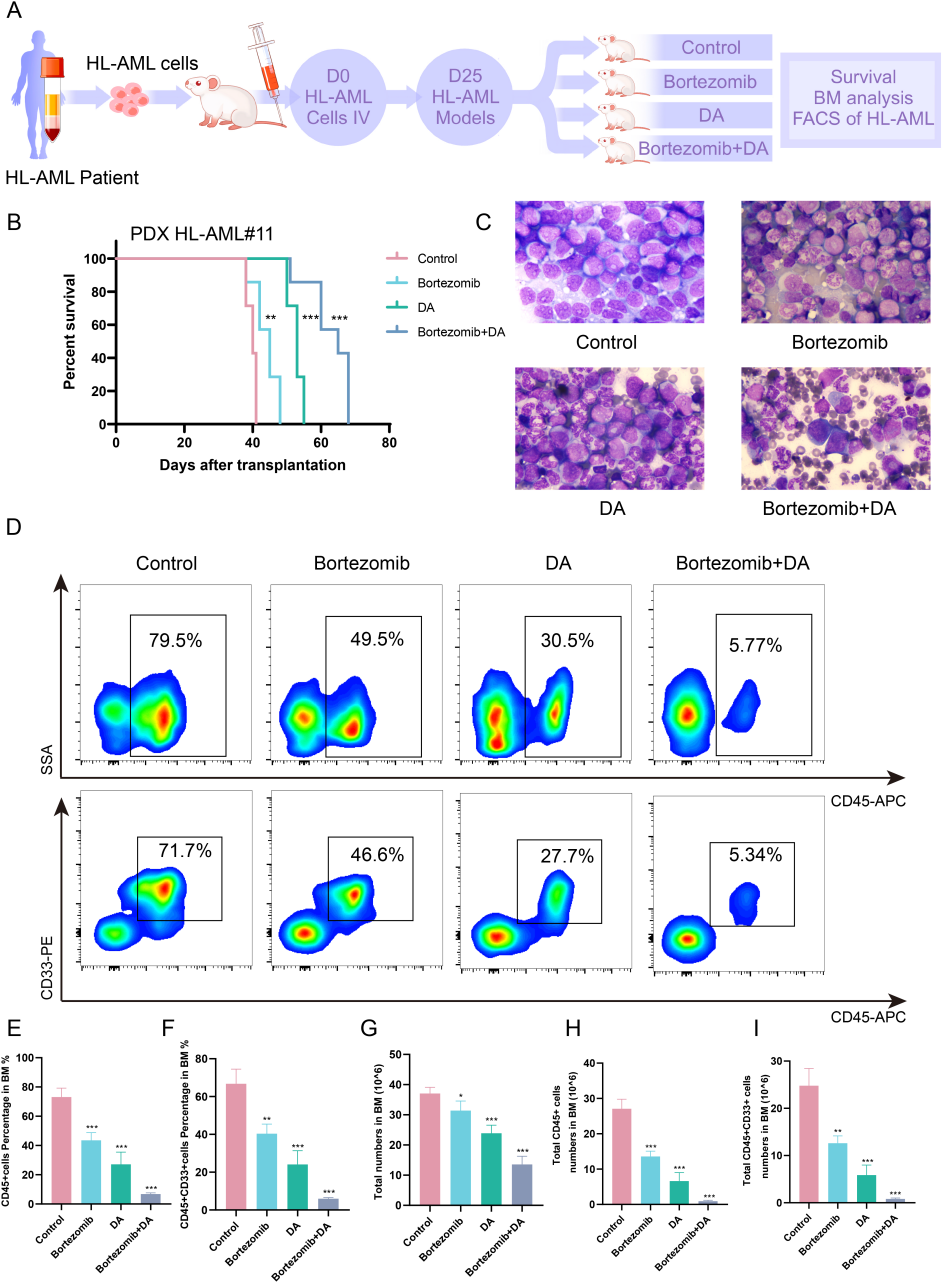
Therapeutic efficacy of bortezomib on the PDX model of HL‐AML. (A) Experimental layout for the intravenous injection of bone marrow cells from HL‐AML patients into NSG mice, establishing an HL‐AML model. (B) Survival analysis. (C) Analysis of bone marrow smears. Magnification fold: × 100 under an oil immersion lens. (D) Flow cytometry analysis of the immunophenotype of leukemia cells in the bone marrow. (E–I) Statistical analysis of the proportion and absolute numbers of leukemia cells. *p < 0.05, **p < 0.01, ***p < 0.001.

In the clinical domain of AML therapeutics, the “3 + 7” chemotherapy regimen, epitomized by the combination of cytarabine and daunorubicin in the DA protocol, has become the standard of induction therapy in this pathology. To ascertain the potential augmentation of efficacy, we probed the synergy between bortezomib and the DA regimen within a constructed PDX model of HL‐AML. NSG mice were intravenously infused with cells derived from HL‐AML patient, subsequently stratified into four cohorts for systematic drug administration (Figure 4A). Treatment with bortezomib in combination with the DA regimen provided a significant survival advantage compared to the other three groups. (Figure 4B). Samples were taken 1 week after drug administration, and a significant reduction of HL‐AML cells was observed in the bortezomib combined with the DA regimen group compared with the other three groups by hematoxylin and eosin staining on bone marrow smears (Figure 4C). Concurrently, discernible reductions in CD45^+^ and CD45^+^ CD33^+^ ratios, along with absolute cell count ratios, were documented in the bortezomib‐combined DA regimen group, indicative of disease remission (Figure 4D–I). These findings posit that the amalgamated therapeutic approach involving bortezomib and the DA regimen exerts a profound and discernible influence on the therapeutic intervention of HL‐AML.

### 3.5 Bortezomib Enhances Therapeutic Efficacy by Suppressing the NF‐Kappa B Pathway

**FIGURE 5:**
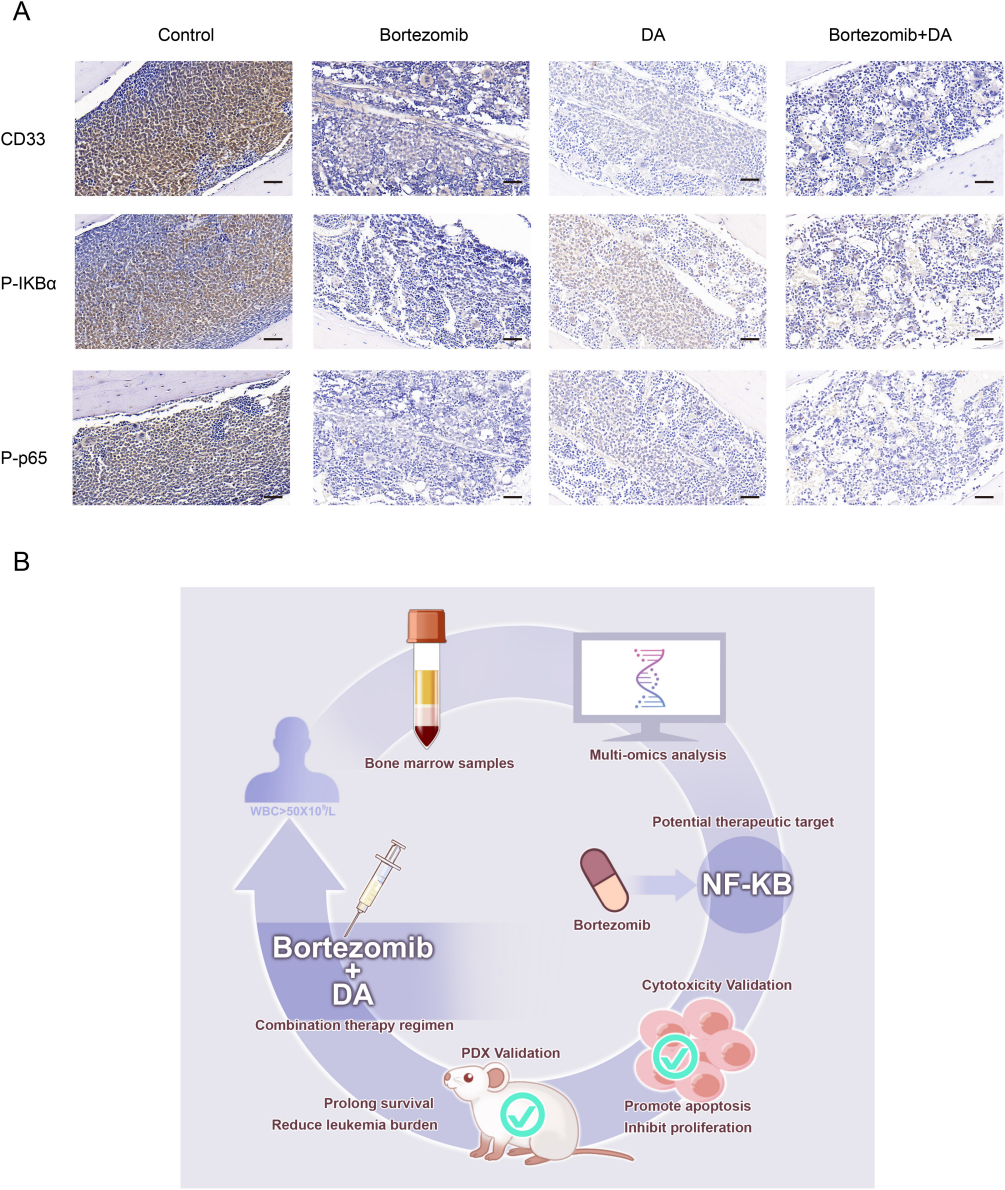
Bortezomib enhances therapeutic efficacy by inhibiting the NF‐kappa B pathway. (A) Immunohistochemical analysis of CD33, P‐IKBα and P‐p65 expression in the PDX model. Scale bar, 50 μm. (B) Schematic diagram illustrating the potential mechanism by which bortezomib enhances the therapeutic efficacy in HL‐AML.

To validate the mechanism of bortezomib, we examined key molecules on the NF‐kappa B pathway, namely, P‐IKBα and P‐p65. The results of bone marrow immunohistochemistry revealed a significant reduction in CD33, P‐IKBα, and P‐p65 in mice treated with the combination of bortezomib and DA regimen (Figure 5A). Therefore, bortezomib enhances the anti‐tumor effect on HL‐AML by suppressing the NF‐kappa B pathway.

## 4 Discussion

Over the past three decades, remarkable advancements have been achieved in the therapeutic landscape of AML, primarily attributable to the introduction of treatment regimens based on anthracycline and cytarabine [15, 16, 17]. However, a significant proportion of patients with AML experience relapse or fail to respond to standard therapy. HL‐AML is a high‐risk type of leukemia with a peripheral blood leukocyte count of 50 × 10^9/L or 100 × 10^9/L. It has an acute onset, is prone to early complications, and, without intervention, can have an early mortality rate of 20% to 40%, requiring urgent treatment. Despite the development of therapeutic modalities, including hydroxyurea, low‐dose chemotherapy, and leukapheresis, current evidence suggests that tailored supportive care has yet to meaningfully impact early mortality rates in HL‐AML patients [18, 19, 20]. A comprehensive understanding of the pathogenic mechanisms governing HL‐AML is crucial to circumvent its occurrence and optimize prognostic outcomes. With the application of high‐throughput molecular biology techniques such as transcriptomics and proteomics, researchers have delved into the intricate BP underpinning cancer. A more exhaustive exploration into the pathogenesis of HL‐AML sets the stage for deeper investigation.

In this study, bone marrow serum from HL‐AML patients was analyzed proteomically using label‐free proteomics. Through comprehensive bioinformatics analyses, we unveiled disparities in protein functionality and pathway distinctions between HL‐AML and NHL‐AML patients. In addition, we analyzed the genetic and functional differences between HL‐AML and NHL‐AML by analyzing the OHSU database. Integrating proteomics and transcriptomics, we identified a significant enrichment of the aberrant NF‐kappa B pathway in HL‐AML. This prompted us to validate whether NF‐kappa B inhibition could represent an effective therapeutic strategy for HL‐AML. So far, the most successful method to inhibit the NF‐kB pathway is to block proteasomal degradation, thereby preventing NF‐kB activation.

Bortezomib is a proteasome inhibitor with broad anti‐tumor activity, exerting its effects by activating multiple signaling cascades, most notably the NF‐kappa B pathway, to induce apoptosis [21]. Clinically, bortezomib is primarily used in the treatment of multiple myeloma and lymphoma [22, 23]. In addition to its role as a proteasome inhibitor, bortezomib also functions as an NF‐kappa B inhibitor. While it demonstrates only modest efficacy as a monotherapy for AML, it is frequently employed in combination with other chemotherapeutic agents for enhanced therapeutic outcomes in leukemia [24, 25]. For instance, bortezomib and belinostat have been shown to act synergistically by suppressing NF‐kappa B activity in leukemia cells [26]. Furthermore, in vivo administration of bortezomib has been demonstrated to inhibit NF‐kappa B activity in leukemia cells [27]. However, scant research has evaluated the therapeutic effects of bortezomib in HL‐AML. Although monotherapy Phase I studies with bortezomib in relapsed/refractory leukemia children indicate limited hematologic improvement, collaborative usage of bortezomib with other compounds demonstrates potent anti‐leukemic activity against AML and suggests potential clinical utility [13, 27]. In addition, the combination of bortezomib, idarubicin, and cytarabine improves overall survival in AML with a favorable safety profile [28].

Hence, further investigation into the combined use of bortezomib and other compounds for AML therapy is warranted. Cytarabine in combination with anthracycline composition regimen is the standard of care for primary AML [29, 30, 31]. We applied bortezomib in combination with DA regimen in a PDX model and surprisingly found that this regimen had a significant killing effect. Additionally, in clinical practice, we administered a combination regimen of bortezomib with DA regimen to two patients diagnosed with HL‐AML. The first patient experienced relapse after five cycles of standard chemotherapy but achieved disease remission following the combination therapy. The second patient, who had previously received multiple chemotherapy regimens without achieving complete remission, attained complete remission after one cycle of the bortezomib–DA combination therapy. Although only two patients have received this regimen so far and further clinical data are required to confirm its efficacy, the remarkable clinical responses observed in these cases provide compelling evidence for conducting more rigorous clinical trials. These trials aim to evaluate bortezomib in combination with the DA regimen as a potential therapeutic strategy for HL‐AML.

Overall, integrated proteomics and transcriptomics revealed the aberrant protein expression profiles and gene expression profiles of HL‐AML and further revealed a significant enrichment of the NF‐kappa B pathway in HL‐AML, which was suggested as a new therapeutic target. The killing effect of bortezomib on HL‐AML cells was verified in vitro and tested in a PDX model with bortezomib in combination with DA regimen (Figure 5B). Our investigation advocates for a viable combinatorial therapeutic approach, bortezomib in combination with DA regimen combination therapeutic strategy, to improve the treatment of HL‐AML.

## 5 Conclusion

In summary, the combination of proteomic and transcriptomic analyses unveiled aberrant protein and gene expression profiles in HL‐AML. Notably, a significant enrichment of the NF‐kappa B pathway in HL‐AML emerged, indicating a novel therapeutic target. Our study also proposes a viable combination therapeutic strategy involving bortezomib in conjunction with the DA regimen to enhance the treatment of HL‐AML.

## Author Contributions

**Jinxian Wu:** formal analysis (equal), project administration (equal), writing – original draft (equal). **Xinqi Li:** project administration (equal), writing – original draft (equal). **Bei Xiong:** project administration (equal), writing – original draft (equal). **Wanyue Yin:** resources (equal), supervision (equal). **Ruihang Li:** resources (equal), supervision (equal). **Guopeng Chen:** data curation (equal), visualization (equal). **Linlu Ma:** data curation (equal), visualization (equal). **Xiqin Tong:** data curation (equal), visualization (equal). **Xiaoyan Liu:** conceptualization (equal), funding acquisition (equal). **Fuling Zhou:** conceptualization (equal), funding acquisition (equal).

## Ethics Statement

The study was approved by the Ethics Committee of Zhongnan Hospital of Wuhan University, and written informed consent forms were obtained from all participants prior to bone marrow puncture.

## Conflicts of Interest

The authors declare no conflicts of interest.

## Supporting information


Data S1.

## Data Availability

The datasets of this study could be obtained from the corresponding author upon reasonable request and found in the publicly OHSU‐AML project (Oregon Health and Science University, accessed through https://www.cbioportal.org/).
