# Supplementary material for: Multi‐Omic Analysis Reveals the Impact of Bortezomib in Hyperleukocytic Acute Myeloid Leukemia
Source: Cancer Med. 2024 Nov 25;13(22):e70438. doi: 10.1002/cam4.70438 (PMC11586862; doi:10.1002/cam4.70438)
Supplement: Supplementary file 1 — Data S1. [file CAM4-13-e70438-s001.docx]

**Supplemental Materials**

**Supplemental Table S1. General information on HL-AML patients and NHL-AML patients.**

| **Patient ID** | **Age** | **Sex** | **category** | **BM blast percentage** | **WBC(10^9/L)** | **FAB** | **Cytogenetics abnormal** | **Surface antigens** |
| --- | --- | --- | --- | --- | --- | --- | --- | --- |
| NHL-AML1 | 69 | Female | NHL-AML | 30.5 | 3.11 | M5 | FLT3-ITD, MLL-PTD | Positive for CD117，CD34，HLA-DR，CD13，CD33; Partial for CD56 |
| NHL-AML2 | 76 | Female | NHL-AML | 33 | 1.03 | M2 | FLT3-ITD, WT1 | Positive for CD117, CD34, CD13, CD33, HLA-DR, CD9, CD123; Partial for CD7, CD64, MPO |
| NHL-AML3 | 38 | Female | NHL-AML | 80.5 | 2.5 | M2 | DEK-NUP214/CAN, WT1 | Positive for CD117, CD13, CD33, HLA-DR, CD38, CD123; Partial for CD34, CD64, MPO |
| NHL-AML4 | 23 | Female | NHL-AML | 50.5 | 1.3 | M2 | MLL-PTD, WT1 | Positive for CD34，CD117，HLA-DR，CD33，CD96，CD13 |
| HL-AML1 | 31 | Female | HL-AML | 86 | 296.47 | M2 | CEBPA-double, WT1 | Positive for CD117, CD13, CD33, HLA-DR; Partial for CD7, CD34, CD38, CD15, CD64, MPO |
| HL-AML2 | 38 | Female | HL-AML | 66.9 | 162 | M5 | FLT3-ITD, CBL, WT1 | Positive for CD117, CD13, CD33, HLA-DR, CD15, CD123; Partial for MPO |
| HL-AML3 | 64 | Female | HL-AML | 83 | 286.2 | M4 | DEK-CAN, WT1 | Positive for CD13, CD33, HLA-DR, CD123; Partial for CD117, CD34, CD38, MPO |
| HL-AML4 | 79 | Male | HL-AML | 91.5 | 150 | M5 | DNMT3A R882H，WT1 | Positive for CD117，CD13，HLA-DR，CD34，CD33; Partial for CD56，CD38，MPO，CD7，CD19，CD64，CD11c |
| HL-AML5 | 75 | Female | HL-AML | 93.5 | 103.9 | M1 | NPM1, HOX11, WT1 | Positive for CD33; Partial for CD117, CD13, CD64, MPO |
| HL-AML6 | 38 | Male | HL-AML | 87 | 330.9 | M2 | FLT3-ITD, CEBPA-double, WT1, EPPK1, TTN, ETV6 | Positive for CD117, CD13, CD33, HLA-DR, CD64; Partial for CD7, CD34, CD38, CD15, MPO |
| HL-AML7 | 82 | Male | HL-AML | 68 | 245 | M2 | DNMT3A R882H,FLT3-ITD, NPM1exon12，WT1 | Positive for CD117，CD13，HLA-DR，CD11c，CD38，CD33; Partial for MPO，CD7，CD71 |
| HL-AML8 | 77 | Male | HL-AML | 95.5 | 233.3 | M1 | FLT3-ITD,NPM1exon12,WT1 | Positive for CD117，CD38，CD33，CD123; Partial for MPO，CD13 |
| HL-AML9 | 52 | Male | HL-AML | 86 | 169.61 | M4 | FLT3-ITD,WT1 | Positive for CD117，CD96，HLA-DR，CD33，CD7，CD64，CD11c; Partial for CD34，CD13，CD11b |
| HL-AML10 | 49 | Male | HL-AML | 78.5 | 287.17 | M5 | DNMT3A R882H,IDH2 R140Q ,WT1 | Positive for HLA-DR，CD117，CD33，CD64，CD13，CD22，CD11c; Partial for CD4，CD34，CD5，CD123，CD2，CD38 |
| NHL-AML5 | 77 | Female | NHL-AML | 76.5 | 2.3 | na | CEBPA-double, WT1 | na |
| NHL-AML6 | 57 | Male | NHL-AML | 85 | 5.09 | na | WT1 | Positive for CD117，CD13，CD38，CD34; Partial for CD123，CD7，HLA-DR |
| NHL-AML7 | 47 | Female | NHL-AML | 82.5 | 5.2 | M2 | WT1 | Positive for CD117，CD13，CD38，CD123，CD56，CD34; Partial for CD5，HLA-DR，CD33， CD64，CD7 |
| HL-AML11 | 63 | Male | HL-AML | 76.5 | 121.82 | M5 | NPM1, WT1 | Positive for HLA-DR，CD11b，CD117，CD38，CD33，CD123; Partial forCD11c，CD7，CD64，MPO |

**Supplemental Table S2. Technical specifications of the antibodies (clones and sources)employed in flow cytometry analysis.**

| **Antibody** | **Conjugate** | **Clone** | **Source** | **Catalog No** | **Dilution** |
| --- | --- | --- | --- | --- | --- |
| anti-human CD45Antibody | APC | HI30 | Biolegend | 304012 | 1/50 |
| anti-human CD33Antibody | PE | WM53 | Biolegend | 303404 | 1/50 |

**Supplemental Table S3. Differentially expressed proteins between HL-AML and NHL-AML.**

| **ID** | logFC | pValue |
| --- | --- | --- |
| SELENOP | -2.16075 | 0.000262206 |
| SEMA7A | -2.179 | 0.000290286 |
| DSC2 | 1.8175 | 0.00050792 |
| COMP | -1.85075 | 0.00052812 |
| ARHGAP1 | 1.4745 | 0.001137914 |
| PI16 | -2.2415 | 0.001189621 |
| DPM3 | -2.193 | 0.001499672 |
| CR2 | -1.344 | 0.001565738 |
| APOA4 | -1.56125 | 0.001622943 |
| CES1 | -1.25025 | 0.002004328 |
| ITGB3 | -1.62575 | 0.002119656 |
| LCP1 | 1.25175 | 0.00238468 |
| FAM118A | -1.5435 | 0.00254053 |
| THBS1 | -2.19225 | 0.002779391 |
| BHMT | -1.72475 | 0.00310152 |
| PLBD2 | 1.32725 | 0.003205302 |
| NUCB1 | 1.44975 | 0.003266264 |
| GLUD1 | 1.40375 | 0.004021683 |
| PPP3CC | -1.3735 | 0.004198246 |
| WDR77 | -1.196 | 0.004269415 |
| ANTXR1 | -1.4525 | 0.004368166 |
| MPO | 1.833 | 0.004572487 |
| F13A1 | -1.73125 | 0.005205958 |
| F13B | -1.770375 | 0.006400711 |
| SPTBN4 | -1.36475 | 0.006791508 |
| GNAI2 | 1.1105 | 0.007182889 |
| MEGF8 | -1.18875 | 0.007285484 |
| SRSF2 | -1.13475 | 0.007367138 |
| CLTB | -1.1375 | 0.007464114 |
| PTK7 | 1.33225 | 0.007703012 |
| VWF | 1.28425 | 0.007947493 |
| GSN | -1.167 | 0.008008816 |
| PPP5C | 1.1345 | 0.008240723 |
| RPL14 | 1.0615 | 0.00847666 |
| MAT2A | 1.424 | 0.008535439 |
| PPBP | -1.37825 | 0.008700492 |
| DCD | -1.6175 | 0.008812309 |
| IRF2BP2 | -2.4185 | 0.008900333 |
| VPS35 | 1.216 | 0.008951079 |
| AZGP1 | -1.0995 | 0.009282605 |
| LBP | 1.307 | 0.009546016 |
| VPS36 | -1.517 | 0.010036372 |
| EHHADH | -1.8465 | 0.010182769 |
| HSP90AB1 | 0.9815 | 0.010242923 |
| MAPK14 | 1.10025 | 0.01032121 |
| HSP90AA1 | 1.09775 | 0.010797178 |
| ADSS2 | 1.234 | 0.010872448 |
| CAPN2 | -0.9825 | 0.010923267 |
| PTPRC | 1.272 | 0.01113797 |
| CD14 | 1.0295 | 0.011195185 |
| RPL7A | 1.20925 | 0.011400611 |
| TNXB | -1.6 | 0.011843527 |
| PDS5A | -1.68725 | 0.012470867 |
| GNB2 | 0.95175 | 0.012474224 |
| NACA | 1.1005 | 0.012574434 |
| PSMD14 | 1.39425 | 0.013362558 |
| RPLP2 | 1.052 | 0.013951298 |
| SRGN | 1.2645 | 0.014531959 |
| IGFBP3 | -1.4305 | 0.014597202 |
| ADAMTS13 | -1.1425 | 0.014715287 |
| ANO9 | -1.275 | 0.014953398 |
| FAU | 1.42625 | 0.015128751 |
| WARS1 | 1.1435 | 0.015417089 |
| F11 | -1.258 | 0.015745636 |
| MBL2 | -1.63925 | 0.016143267 |
| TTC9 | -1.235 | 0.0163245 |
| SERPINA7 | -1.3885 | 0.016671052 |
| CALR | 1.07825 | 0.016718751 |
| MTAP | 1.234 | 0.016725225 |
| G3BP1 | 1.31925 | 0.017171288 |
| CGNL1 | -0.9965 | 0.017517511 |
| ADGRG2 | -1.22375 | 0.017533976 |
| RAP1A | 0.8675 | 0.017993574 |
| HSPA4 | 1.0865 | 0.018374609 |
| NAGLU | -1.03475 | 0.018478879 |
| SPARC | -1.09575 | 0.018714852 |
| CD82 | 0.91575 | 0.018744827 |
| RFC1 | -1.062 | 0.019385422 |
| THBS4 | -2.0165 | 0.019927921 |
| ALB | -1.017 | 0.021122353 |
| DBNL | -1.03025 | 0.02138155 |
| GPX3 | -1.07575 | 0.021774312 |
| ATRN | -1.00575 | 0.022026292 |
| AFM | -1.0575 | 0.022675834 |
| LUM | -1.315 | 0.023681155 |
| SNX5 | 0.87225 | 0.02458195 |
| RPS6 | 1.17575 | 0.02486229 |
| SYK | 2.003 | 0.02497716 |
| ICAM1 | -0.82225 | 0.024987021 |
| IGHG4 | -1.16275 | 0.025084179 |
| XPNPEP1 | 0.91425 | 0.025835055 |
| CLU | -1.07725 | 0.026577767 |
| UGP2 | 1.313 | 0.02717388 |
| DSP | -1.071 | 0.027414385 |
| LCAT | -1.05275 | 0.027828319 |
| CRTAC1 | -1.62475 | 0.029249169 |
| MMP9 | -0.94225 | 0.029354506 |
| RPS2 | 0.99325 | 0.02981341 |
| IGFALS | -1.0875 | 0.030730254 |
| APOA2 | -0.98175 | 0.030873209 |
| SERPINA6 | -0.84725 | 0.031017917 |
| RNPEP | 1.169 | 0.031090978 |
| HEXB | 1.131 | 0.031683092 |
| NRBP1 | 0.863 | 0.031963583 |
| CAVIN2 | -1.09875 | 0.032526315 |
| RPS15A | 0.94625 | 0.032620811 |
| CAB39 | 0.856 | 0.033216348 |
| FBLN1 | -1.134 | 0.033258423 |
| IGF2 | -1.19525 | 0.033733009 |
| PLIN3 | 0.9395 | 0.03380746 |
| GNB1 | 1.0485 | 0.034132548 |
| MAP3K21 | -0.8835 | 0.034134097 |
| MAPK1 | 0.94675 | 0.034424036 |
| PCBD1 | -1.164 | 0.034508678 |
| SPP2 | -1.03975 | 0.034609827 |
| ARL6IP5 | 0.91925 | 0.034889023 |
| IGHG1 | -1.56025 | 0.035169234 |
| VAT1 | 1.3555 | 0.035768398 |
| MZB1 | -0.88925 | 0.035843845 |
| NEDD8-MDP1 | -0.7925 | 0.035872638 |
| MAN2B1 | 1.52175 | 0.036628081 |
| DNPEP | 1.089 | 0.036756807 |
| YWHAH | 0.88575 | 0.037865357 |
| GPLD1 | -1.0375 | 0.038469358 |
| CNTN1 | -0.85175 | 0.038617137 |
| MENT | -1.51425 | 0.038972921 |
| MYOC | -1.2715 | 0.039143946 |
| TPR | -0.909 | 0.040197636 |
| VSIG4 | 1.06625 | 0.040243408 |
| FLNA | -0.9535 | 0.040263492 |
| SORL1 | 1.6575 | 0.04060652 |
| FETUB | -1.38275 | 0.040788962 |
| ADIPOQ | -1.08475 | 0.041036886 |
| FGFBP2 | -0.91525 | 0.04171878 |
| CCDC93 | -0.9005 | 0.041851833 |
| ENPEP | -0.8275 | 0.043185462 |
| KLKB1 | -1.0165 | 0.043346945 |
| AHNAK | 1.05475 | 0.044057906 |
| MSN | 0.79325 | 0.044304143 |
| KARS1 | 0.966 | 0.044519204 |
| ADGRE5 | 1.44525 | 0.044716564 |
| FH | -0.84325 | 0.044966214 |
| RBP4 | -1.3245 | 0.044978832 |
| DRG1 | 0.9155 | 0.04517902 |
| NPC2 | 1.09775 | 0.045294062 |
| VCAM1 | 0.8065 | 0.04543386 |
| PARP1 | 0.80625 | 0.046256649 |
| ENO1 | 0.9235 | 0.046519286 |
| NPEPPS | 1.8715 | 0.046912398 |
| IL32 | -0.74925 | 0.046964445 |
| DEFB1 | -0.81275 | 0.047268541 |
| UPF1 | 0.8115 | 0.047523856 |
| RPS24 | 0.89525 | 0.047693007 |
| CANX | 1.0385 | 0.048224946 |
| KNG1 | -0.839 | 0.048677365 |
| PLXNB1 | -1.09225 | 0.049490234 |
| PCDH18 | -1.47325 | 0.049729384 |
| GC | -1.046125 | 0.049738314 |
| H2BC18 | 2.03375 | 0.049956967 |
| MRC1 | 0.91425 | 0.049987034 |

**Supplemental Table S4. Top 10 Gene Ontology (GO) enrichment terms for differentially expressed proteins in HL-AML and NHL-AML, categorized into biological process (BP), cellular component (CC), and molecular function (MF).**

| ONTOLOGY | ID | Description | GeneRatio | BgRatio | pvalue | p.adjust | qvalue | geneID | Count |
| --- | --- | --- | --- | --- | --- | --- | --- | --- | --- |
| BP | GO:0007596 | blood coagulation | 14/155 | 221/18800 | 4.27836E-09 | 6.19822E-06 | 4.76257E-06 | COMP/ITGB3/THBS1/F13A1/F13B/VWF/MAPK14/ADAMTS13/F11/SYK/FBLN1/FLNA/KLKB1/KNG1 | 14 |
| BP | GO:0050817 | coagulation | 14/155 | 226/18800 | 5.70313E-09 | 6.19822E-06 | 4.76257E-06 | COMP/ITGB3/THBS1/F13A1/F13B/VWF/MAPK14/ADAMTS13/F11/SYK/FBLN1/FLNA/KLKB1/KNG1 | 14 |
| BP | GO:0007599 | hemostasis | 14/155 | 227/18800 | 6.03527E-09 | 6.19822E-06 | 4.76257E-06 | COMP/ITGB3/THBS1/F13A1/F13B/VWF/MAPK14/ADAMTS13/F11/SYK/FBLN1/FLNA/KLKB1/KNG1 | 14 |
| BP | GO:0043691 | reverse cholesterol transport | 5/155 | 20/18800 | 5.00985E-07 | 0.000330718 | 0.000254116 | APOA4/CES1/CLU/LCAT/APOA2 | 5 |
| BP | GO:0042060 | wound healing | 16/155 | 429/18800 | 5.36705E-07 | 0.000330718 | 0.000254116 | COMP/ITGB3/THBS1/F13A1/F13B/PTK7/VWF/MAPK14/ADAMTS13/F11/SYK/DSP/FBLN1/FLNA/KLKB1/KNG1 | 16 |
| BP | GO:0050878 | regulation of body fluid levels | 15/155 | 382/18800 | 6.50501E-07 | 0.000334032 | 0.000256662 | COMP/ITGB3/THBS1/F13A1/F13B/GNAI2/VWF/MAPK14/ADAMTS13/F11/SYK/FBLN1/FLNA/KLKB1/KNG1 | 15 |
| BP | GO:0006909 | phagocytosis | 13/155 | 310/18800 | 1.8208E-06 | 0.000801412 | 0.000615786 | ITGB3/THBS1/GSN/LBP/PTPRC/CD14/MBL2/CALR/SYK/IGHG4/APOA2/IGHG1/ADIPOQ | 13 |
| BP | GO:0051604 | protein maturation | 13/155 | 314/18800 | 2.09804E-06 | 0.000808007 | 0.000620853 | COMP/THBS1/GSN/CAPN2/SRGN/ADAMTS13/F11/CALR/SORL1/ENPEP/KLKB1/PARP1/ENO1 | 13 |
| BP | GO:1902947 | regulation of tau-protein kinase activity | 4/155 | 14/18800 | 4.17211E-06 | 0.001356283 | 0.001042136 | HSP90AB1/HSP90AA1/CLU/SORL1 | 4 |
| BP | GO:0010876 | lipid localization | 15/155 | 446/18800 | 4.40209E-06 | 0.001356283 | 0.001042136 | APOA4/CES1/ITGB3/THBS1/LBP/ANO9/SYK/CLU/LCAT/APOA2/HEXB/PLIN3/ADIPOQ/RBP4/NPC2 | 15 |
| CC | GO:0034774 | secretory granule lumen | 25/156 | 322/19594 | 7.97348E-18 | 1.24365E-15 | 9.05061E-16 | SELENOP/THBS1/MPO/F13A1/VWF/GSN/PPBP/HSP90AB1/MAPK14/HSP90AA1/PSMD14/SRGN/SPARC/ALB/DBNL/CLU/HEXB/CAB39/IGF2/MAPK1/SPP2/VAT1/MAN2B1/NPC2/KNG1 | 25 |
| CC | GO:0060205 | cytoplasmic vesicle lumen | 25/156 | 325/19594 | 9.94851E-18 | 1.24365E-15 | 9.05061E-16 | SELENOP/THBS1/MPO/F13A1/VWF/GSN/PPBP/HSP90AB1/MAPK14/HSP90AA1/PSMD14/SRGN/SPARC/ALB/DBNL/CLU/HEXB/CAB39/IGF2/MAPK1/SPP2/VAT1/MAN2B1/NPC2/KNG1 | 25 |
| CC | GO:0031983 | vesicle lumen | 25/156 | 327/19594 | 1.15153E-17 | 1.24365E-15 | 9.05061E-16 | SELENOP/THBS1/MPO/F13A1/VWF/GSN/PPBP/HSP90AB1/MAPK14/HSP90AA1/PSMD14/SRGN/SPARC/ALB/DBNL/CLU/HEXB/CAB39/IGF2/MAPK1/SPP2/VAT1/MAN2B1/NPC2/KNG1 | 25 |
| CC | GO:0062023 | collagen-containing extracellular matrix | 22/156 | 429/19594 | 4.05446E-12 | 3.28412E-10 | 2.39E-10 | SEMA7A/COMP/APOA4/THBS1/F13A1/VWF/AZGP1/TNXB/MBL2/CALR/SPARC/THBS4/ATRN/LUM/ICAM1/CLU/MMP9/FBLN1/SPP2/MYOC/ADIPOQ/KNG1 | 22 |
| CC | GO:0031093 | platelet alpha granule lumen | 10/156 | 67/19594 | 1.28607E-10 | 8.02234E-09 | 5.83822E-09 | THBS1/F13A1/VWF/PPBP/SRGN/SPARC/ALB/CLU/IGF2/KNG1 | 10 |
| CC | GO:0031091 | platelet alpha granule | 11/156 | 91/19594 | 1.56335E-10 | 8.02234E-09 | 5.83822E-09 | ITGB3/THBS1/F13A1/VWF/PPBP/SRGN/SPARC/ALB/CLU/IGF2/KNG1 | 11 |
| CC | GO:0072562 | blood microparticle | 13/156 | 147/19594 | 1.73322E-10 | 8.02234E-09 | 5.83822E-09 | APOA4/F13A1/GSN/ALB/AFM/IGHG4/CLU/APOA2/IGHG1/DNPEP/MSN/KNG1/GC | 13 |
| CC | GO:0005925 | focal adhesion | 18/156 | 419/19594 | 6.88231E-09 | 2.78733E-07 | 2.02847E-07 | ITGB3/LCP1/PTK7/GSN/CAPN2/PTPRC/RPL7A/GNB2/RPLP2/CALR/G3BP1/ICAM1/RPS2/MAPK1/FLNA/AHNAK/MSN/ADGRE5 | 18 |
| CC | GO:0030055 | cell-substrate junction | 18/156 | 428/19594 | 9.56799E-09 | 3.44448E-07 | 2.5067E-07 | ITGB3/LCP1/PTK7/GSN/CAPN2/PTPRC/RPL7A/GNB2/RPLP2/CALR/G3BP1/ICAM1/RPS2/MAPK1/FLNA/AHNAK/MSN/ADGRE5 | 18 |
| CC | GO:0005788 | endoplasmic reticulum lumen | 15/156 | 311/19594 | 2.93878E-08 | 9.52164E-07 | 6.92933E-07 | APOA4/CES1/THBS1/NUCB1/IGFBP3/ADAMTS13/CALR/ALB/CLU/APOA2/MAPK1/SPP2/MZB1/CANX/KNG1 | 15 |
| MF | GO:0005178 | integrin binding | 15/155 | 156/18410 | 4.14203E-12 | 1.57397E-09 | 1.23825E-09 | SEMA7A/COMP/ITGB3/LCP1/THBS1/VWF/TNXB/ADAMTS13/CALR/THBS4/SYK/ICAM1/FBLN1/IGF2/VCAM1 | 15 |
| MF | GO:0005518 | collagen binding | 8/155 | 68/18410 | 1.01495E-07 | 1.92841E-05 | 1.51709E-05 | COMP/THBS1/ANTXR1/VWF/TNXB/SPARC/LUM/MMP9 | 8 |
| MF | GO:0004177 | aminopeptidase activity | 6/155 | 39/18410 | 8.38326E-07 | 0.000106188 | 8.35385E-05 | F11/XPNPEP1/RNPEP/DNPEP/ENPEP/NPEPPS | 6 |
| MF | GO:0001968 | fibronectin binding | 5/155 | 31/18410 | 5.6461E-06 | 0.000536379 | 0.000421972 | ITGB3/THBS1/IGFBP3/FBLN1/MYOC | 5 |
| MF | GO:0070006 | metalloaminopeptidase activity | 4/155 | 27/18410 | 7.29436E-05 | 0.005543712 | 0.004361258 | XPNPEP1/RNPEP/ENPEP/NPEPPS | 4 |
| MF | GO:0008201 | heparin binding | 8/155 | 168/18410 | 8.98061E-05 | 0.005687719 | 0.004474549 | COMP/THBS1/MPO/PTPRC/TNXB/F11/THBS4/KNG1 | 8 |
| MF | GO:0005201 | extracellular matrix structural constituent | 8/155 | 172/18410 | 0.000105808 | 0.005743841 | 0.004518701 | COMP/THBS1/VWF/TNXB/SPARC/LUM/FBLN1/ADIPOQ | 8 |
| MF | GO:0045296 | cadherin binding | 11/155 | 333/18410 | 0.000122789 | 0.005832458 | 0.004588416 | ARHGAP1/RPL14/HSP90AB1/RPL7A/DBNL/SNX5/RPS2/PLIN3/FLNA/AHNAK/ENO1 | 11 |
| MF | GO:0008237 | metallopeptidase activity | 8/155 | 189/18410 | 0.000202481 | 0.008503887 | 0.006690039 | PSMD14/ADAMTS13/XPNPEP1/MMP9/RNPEP/DNPEP/ENPEP/NPEPPS | 8 |
| MF | GO:0008238 | exopeptidase activity | 6/155 | 102/18410 | 0.000223787 | 0.008503887 | 0.006690039 | F11/XPNPEP1/RNPEP/DNPEP/ENPEP/NPEPPS | 6 |

**Supplemental Table S5. Top 10 Gene Ontology (GO) enrichment terms for differentially expressed genes in HL-AML and NHL-AML, categorized into biological process (BP), cellular component (CC), and molecular function (MF) in the OHSU-AML cohort.**

| ONTOLOGY | ID | Description | GeneRatio | BgRatio | pvalue | p.adjust | qvalue | geneID | Count |
| --- | --- | --- | --- | --- | --- | --- | --- | --- | --- |
| BP | GO:0050867 | positive regulation of cell activation | 91/981 | 467/18903 | 1.26E-28 | 6.81E-25 | 5.21E-25 | VNN1/PIK3R6/LGALS1/PYCARD/ACTA2/TNFSF4/VAMP8/TNFSF13/ANXA1/BTK/CD160/IRS2/IGHV3-30/IGHV2-5/F2RL1/FOXP3/IGHV1-69/RHOH/TGFBR2/FYN/IGHV3-74/IGHV3-48/GP1BA/IL6/IGHV4-39/IGLC7/IGHV3-33/TNF/CD274/TNFRSF14/IGHD/FCRL3/CLEC4D/MAP3K8/PDGFRB/TMIGD2/IGHV5-51/GP9/TRDC/HLA-F/IGHM/IGHV3-15/IL6ST/SIRPG/PELI1/SLAMF1/ICOSLG/CD83/CARD11/CCL2/CCL5/DPP4/IGHV3-21/MDK/CD40LG/AXL/IGHV3-7/VCAM1/IGLC2/IGHV3-23/CD177/CD27/RUNX3/CD2/CD226/IGHG4/IGHA2/THBS1/GATA1/SPTA1/IGLC3/IGHG3/EGR3/IGHG2/CCR7/TRBC2/TBX21/LCK/GATA3/IGHA1/ICOS/IGHG1/CD28/RASGRP1/IL7R/NR4A3/CD3E/LEF1/ZAP70/CD5/CD6 | 91 |
| BP | GO:0050851 | antigen receptor-mediated signaling pathway | 65/981 | 252/18903 | 6.72E-28 | 1.82E-24 | 1.39E-24 | TRGC2/LPXN/BTK/FCGR2B/CD160/TNFRSF21/SLA2/PLEKHA1/IGHV3-30/IGHV2-5/FOXP3/IGHV1-69/FYN/IGHV3-74/IGHV3-48/IGHV4-39/IGLC7/IGHV3-33/PTPRJ/IGHD/FCRL3/IGHV5-51/TRDC/IGHM/IGHV3-15/PDE4D/ZC3H12A/ICOSLG/CARD11/IGHV3-21/SKAP1/TRAT1/IGHV3-7/CEACAM1/CTLA4/IGLC2/IGHV3-23/PRKCH/THEMIS/GBP1/CD8B/CD3G/UBASH3A/PLCG1/CD226/IGHG4/IGHA2/IGLC3/IGHG3/IGHG2/CCR7/TRAC/PDE4B/TRBC2/LCK/GATA3/CD3D/IGHA1/IGHG1/CD28/CD3E/CD247/ZAP70/ITK/CD8A | 65 |
| BP | GO:0002460 | adaptive immune response based on somatic recombination of immune receptors built from immunoglobulin superfamily domains | 79/981 | 375/18903 | 2.77E-27 | 4.99E-24 | 3.81E-24 | GAPT/CARD9/P2RX7/TNFSF4/FUT7/SPN/UNG/C5/TNFSF13/ANXA1/BTK/FCGR2B/PVR/NFKB2/HLA-B/SLA2/IGHV3-30/RSAD2/IGHV2-5/FOXP3/IGHV1-69/ARID5A/C1QB/IGHV3-74/IGHV3-48/IL6/IGHV4-39/LTA/IGLC7/CSF2RB/IGHV3-33/TNF/CD274/BCL3/IGHD/IGHV5-51/TRDC/HLA-F/IGHM/IGHV3-15/RELB/ICAM1/BACH2/ZC3H12A/SLAMF1/IRF4/ARG1/IGHV3-21/IL21R/IL1R1/CD40LG/IGHV3-7/TNFAIP3/CEACAM1/KLRD1/LY9/IGLC2/SMAD7/IGHV3-23/CD27/GZMM/IL18R1/CD226/IGHG4/IGHA2/IGLC3/IGHG3/IGHG2/PRF1/TRBC2/TBX21/GATA3/IGHA1/IGHG1/CD28/IL7R/LEF1/CD8A/RORA | 79 |
| BP | GO:0002696 | positive regulation of leukocyte activation | 87/981 | 450/18903 | 3.85E-27 | 5.21E-24 | 3.99E-24 | VNN1/PIK3R6/LGALS1/PYCARD/TNFSF4/VAMP8/TNFSF13/ANXA1/BTK/CD160/IRS2/IGHV3-30/IGHV2-5/F2RL1/FOXP3/IGHV1-69/RHOH/TGFBR2/FYN/IGHV3-74/IGHV3-48/IL6/IGHV4-39/IGLC7/IGHV3-33/TNF/CD274/TNFRSF14/IGHD/FCRL3/CLEC4D/MAP3K8/TMIGD2/IGHV5-51/TRDC/HLA-F/IGHM/IGHV3-15/IL6ST/SIRPG/PELI1/SLAMF1/ICOSLG/CD83/CARD11/CCL2/CCL5/DPP4/IGHV3-21/MDK/CD40LG/AXL/IGHV3-7/VCAM1/IGLC2/IGHV3-23/CD177/CD27/RUNX3/CD2/CD226/IGHG4/IGHA2/THBS1/GATA1/SPTA1/IGLC3/IGHG3/EGR3/IGHG2/CCR7/TRBC2/TBX21/LCK/GATA3/IGHA1/ICOS/IGHG1/CD28/RASGRP1/IL7R/NR4A3/CD3E/LEF1/ZAP70/CD5/CD6 | 87 |
| BP | GO:0002449 | lymphocyte mediated immunity | 77/981 | 367/18903 | 1.71E-26 | 1.85E-23 | 1.41E-23 | GAPT/CARD9/PIK3R6/P2RX7/TNFSF4/FUT7/UNG/C5/TNFSF13/BTK/FCGR2B/PVR/CD160/HLA-B/SLA2/IGHV3-30/RSAD2/IGHV2-5/FOXP3/IGHV1-69/ARID5A/C1QB/IGHV3-74/IGHV3-48/IL6/IGHV4-39/LTA/IGLC7/CSF2RB/IGHV3-33/TNF/BCL3/SERPINB9/IGHD/IGHV5-51/TRDC/HLA-F/IGHM/IGHV3-15/ICAM1/LAG3/SH2D1B/SLAMF1/ARG1/IGHV3-21/IL21R/IL1R1/CD40LG/IGHV3-7/CEACAM1/SLAMF7/KLRD1/IGLC2/SMAD7/IGHV3-23/CD27/GZMM/IL18R1/CD2/CD226/IGHG4/IGHA2/IGLC3/IGHG3/KLRB1/IGHG2/PRF1/TRBC2/TBX21/GATA3/IGHA1/GZMB/IGHG1/CD28/RASGRP1/IL7R/CD8A | 77 |
| BP | GO:0002253 | activation of immune response | 79/981 | 397/18903 | 1.44E-25 | 1.11E-22 | 8.53E-23 | CFD/TRGC2/PYCARD/LPXN/C5/BTK/FCGR2B/CD160/TNFRSF21/SLA2/PLEKHA1/IGHV3-30/ZNFX1/IGHV2-5/FOXP3/IGHV1-69/C1QB/FFAR2/FYN/IGHV3-74/IGHV3-48/IGHV4-39/IGLC7/IGHV3-33/PTPRJ/IGHD/FCRL3/KRT1/IGHV5-51/TRDC/IGHM/CD5L/A2M/IGHV3-15/PDE4D/ZC3H12A/ICOSLG/CARD11/IGHV3-21/SKAP1/TRAT1/IGHV3-7/CEACAM1/KLRD1/CTLA4/IGLC2/IGHV3-23/PRKCH/THEMIS/GBP1/CD8B/PYHIN1/PTK2/CD3G/UBASH3A/PLCG1/CD226/IGHG4/IGHA2/ZBP1/IGLC3/IGHG3/IGHG2/CCR7/TRAC/PDE4B/TRBC2/LCK/GATA3/CD3D/IGHA1/IGHG1/CD28/NR4A3/CD3E/CD247/ZAP70/ITK/CD8A | 79 |
| BP | GO:0002443 | leukocyte mediated immunity | 86/981 | 463/18903 | 1.44E-25 | 1.11E-22 | 8.53E-23 | GAPT/CARD9/PIK3R6/P2RX7/TNFSF4/FUT7/VAMP8/UNG/C5/TNFSF13/BTK/FCGR2B/PVR/CD160/HLA-B/SLA2/IGHV3-30/RSAD2/IGHV2-5/F2RL1/PTGDR/FOXP3/IGHV1-69/ARID5A/C1QB/IGHV3-74/IGHV3-48/IL6/IGHV4-39/LTA/IGLC7/CSF2RB/IGHV3-33/TNF/BCL3/NLRP6/SERPINB9/IGHD/IGHV5-51/TRDC/HLA-F/IGHM/PTGDS/IGHV3-15/ICAM1/LAG3/SH2D1B/SLAMF1/ARG1/IGHV3-21/IL21R/ANXA3/IL1R1/CD40LG/IGHV3-7/CEACAM1/SLAMF7/KLRD1/IGLC2/SMAD7/IGHV3-23/CD177/CD27/GZMM/IL18R1/CD2/CD226/IGHG4/IGHA2/GATA1/IGLC3/IGHG3/KLRB1/IGHG2/PRF1/TRBC2/TBX21/GATA3/IGHA1/GZMB/IGHG1/CD28/RASGRP1/IL7R/NR4A3/CD8A | 86 |
| BP | GO:0002429 | immune response-activating cell surface receptor signaling pathway | 69/981 | 312/18903 | 2.99E-25 | 1.80E-22 | 1.37E-22 | TRGC2/LPXN/BTK/FCGR2B/CD160/TNFRSF21/SLA2/PLEKHA1/IGHV3-30/IGHV2-5/FOXP3/IGHV1-69/FFAR2/FYN/IGHV3-74/IGHV3-48/IGHV4-39/IGLC7/IGHV3-33/PTPRJ/IGHD/FCRL3/IGHV5-51/TRDC/IGHM/IGHV3-15/PDE4D/ZC3H12A/ICOSLG/CARD11/IGHV3-21/SKAP1/TRAT1/IGHV3-7/CEACAM1/KLRD1/CTLA4/IGLC2/IGHV3-23/PRKCH/THEMIS/GBP1/CD8B/PTK2/CD3G/UBASH3A/PLCG1/CD226/IGHG4/IGHA2/IGLC3/IGHG3/IGHG2/CCR7/TRAC/PDE4B/TRBC2/LCK/GATA3/CD3D/IGHA1/IGHG1/CD28/NR4A3/CD3E/CD247/ZAP70/ITK/CD8A | 69 |
| BP | GO:0002757 | immune response-activating signal transduction | 69/981 | 312/18903 | 2.99E-25 | 1.80E-22 | 1.37E-22 | TRGC2/LPXN/BTK/FCGR2B/CD160/TNFRSF21/SLA2/PLEKHA1/IGHV3-30/IGHV2-5/FOXP3/IGHV1-69/FFAR2/FYN/IGHV3-74/IGHV3-48/IGHV4-39/IGLC7/IGHV3-33/PTPRJ/IGHD/FCRL3/IGHV5-51/TRDC/IGHM/IGHV3-15/PDE4D/ZC3H12A/ICOSLG/CARD11/IGHV3-21/SKAP1/TRAT1/IGHV3-7/CEACAM1/KLRD1/CTLA4/IGLC2/IGHV3-23/PRKCH/THEMIS/GBP1/CD8B/PTK2/CD3G/UBASH3A/PLCG1/CD226/IGHG4/IGHA2/IGLC3/IGHG3/IGHG2/CCR7/TRAC/PDE4B/TRBC2/LCK/GATA3/CD3D/IGHA1/IGHG1/CD28/NR4A3/CD3E/CD247/ZAP70/ITK/CD8A | 69 |
| BP | GO:0051251 | positive regulation of lymphocyte activation | 77/981 | 400/18903 | 5.46E-24 | 2.95E-21 | 2.26E-21 | VNN1/PIK3R6/LGALS1/PYCARD/TNFSF4/TNFSF13/ANXA1/BTK/CD160/IRS2/IGHV3-30/IGHV2-5/FOXP3/IGHV1-69/RHOH/TGFBR2/FYN/IGHV3-74/IGHV3-48/IL6/IGHV4-39/IGLC7/IGHV3-33/CD274/TNFRSF14/IGHD/FCRL3/MAP3K8/TMIGD2/IGHV5-51/TRDC/HLA-F/IGHM/IGHV3-15/IL6ST/SIRPG/PELI1/SLAMF1/ICOSLG/CD83/CARD11/CCL2/CCL5/DPP4/IGHV3-21/MDK/CD40LG/AXL/IGHV3-7/VCAM1/IGLC2/IGHV3-23/CD27/RUNX3/IGHG4/IGHA2/SPTA1/IGLC3/IGHG3/EGR3/IGHG2/CCR7/TRBC2/TBX21/LCK/GATA3/IGHA1/ICOS/IGHG1/CD28/RASGRP1/IL7R/CD3E/LEF1/ZAP70/CD5/CD6 | 77 |
| CC | GO:0009897 | external side of plasma membrane | 100/1016 | 462/19869 | 6.49E-36 | 3.58E-33 | 3.10E-33 | SCUBE1/CD33/TRGC2/TCN2/P2RX7/CD9/SPN/GPC4/IL3RA/ANXA1/CSF2RA/FCGR2B/IGHV3-30/IGHV2-5/IGHV1-69/TGFBR2/IGHV3-74/CD69/IGHV3-48/GP1BA/IGHV4-39/IGLC7/CSF2RB/IGHV3-33/TNF/CD274/TNFRSF14/FCGRT/IGHD/SLC7A5/CLEC4D/CA4/IGHV5-51/TRDC/HLA-F/IGHM/PDCD1/CXCR1/IGHV3-15/ICAM1/IL6ST/LAG3/SLAMF1/CXCR5/ICOSLG/ATP1B2/CD83/CXCR3/EPOR/ECE1/HEG1/IGHV3-21/ABCG1/CD48/IL21R/IL1R1/FCRL6/CD40LG/SEMA7A/IGHV3-7/ABCA1/MCAM/VCAM1/SLAMF7/KLRD1/CTLA4/LY9/IGLC2/IGHV3-23/CCR4/CD27/ITGA2B/CD3G/THBD/ITGB3/CD2/CD34/CD226/IGHG4/IGHA2/THBS1/IGLC3/IGHG3/ABCB1/IGHG2/CCR7/TRBC2/CD3D/CXCL12/IGHA1/S1PR1/IGHG1/CD28/IL7R/CD3E/TGFBR3/CD5/CD8A/CD6/IL2RB | 100 |
| CC | GO:0019814 | immunoglobulin complex | 42/1016 | 166/19869 | 2.45E-18 | 6.75E-16 | 5.85E-16 | IGKV1-9/IGHV3-30/IGHV2-5/IGHV1-69/IGHV3-74/IGHV3-48/IGHV4-39/IGLV3-25/IGLV1-47/IGLV3-19/IGLC7/IGHV3-33/IGLV6-57/IGKV3-15/IGHD/IGHV5-51/IGLV3-1/IGLV1-44/TRDC/IGLV2-11/IGHM/IGLV2-23/IGHV3-15/IGLV3-21/IGLV2-8/IGLV1-40/IGHV3-21/IGHV3-7/IGKV3-20/IGKV1-5/IGLC2/IGHV3-23/IGLV2-14/IGKV4-1/IGHG4/IGHA2/IGLC3/IGHG3/IGHG2/TRBC2/IGHA1/IGHG1 | 42 |
| CC | GO:0042571 | immunoglobulin complex, circulating | 26/1016 | 76/19869 | 2.58E-15 | 4.74E-13 | 4.10E-13 | IGHV3-30/IGHV2-5/IGHV1-69/IGHV3-74/IGHV3-48/IGHV4-39/IGLC7/IGHV3-33/IGHD/IGHV5-51/TRDC/IGHM/IGHV3-15/IGHV3-21/IGHV3-7/IGKV3-20/IGLC2/IGHV3-23/IGHG4/IGHA2/IGLC3/IGHG3/IGHG2/TRBC2/IGHA1/IGHG1 | 26 |
| CC | GO:0072562 | blood microparticle | 35/1016 | 145/19869 | 7.88E-15 | 1.09E-12 | 9.39E-13 | HSPA1B/C1QB/IGLV3-25/IGLV1-47/STOM/IGKV3-15/IGHD/KRT1/IGHM/CD5L/A2M/IGLV3-21/ORM1/SLC2A1/IGHV3-7/IGKV3-20/IGKV1-5/IGLC2/IGHV3-23/ITGA2B/IGKV4-1/IGHG4/IGHA2/IGLC3/IGHG3/APOE/IGHG2/IGHA1/HBD/IGHG1/HBG2/SLC4A1/HBA1/HBA2/HBB | 35 |
| CC | GO:0042581 | specific granule | 33/1016 | 160/19869 | 5.11E-12 | 5.63E-10 | 4.87E-10 | ATP8B4/CD33/LAIR1/CLEC12A/AOC1/VAMP8/GPR84/PTPRJ/SLC2A3/STOM/CKAP4/CHIT1/CLEC4D/TCN1/CEACAM3/ORM1/ARG1/OLR1/ANXA3/FCAR/QPCT/CEACAM1/CXCL1/SLC44A2/PGLYRP1/CD177/MMP25/CRISP3/OLFM4/CAMP/CHI3L1/LCN2/LTF | 33 |
| CC | GO:0005833 | hemoglobin complex | 9/1016 | 12/19869 | 4.42E-10 | 4.06E-08 | 3.51E-08 | HBQ1/HBG1/HBM/HBD/HBG2/AHSP/HBA1/HBA2/HBB | 9 |
| CC | GO:0070820 | tertiary granule | 30/1016 | 164/19869 | 1.03E-09 | 8.07E-08 | 6.98E-08 | IDH1/ATP8B4/CD33/METTL7A/LAIR1/DYNLL1/CLEC12A/VAMP8/GPR84/SLC2A3/STOM/CHIT1/CLEC4D/TCN1/MGAM/ORM1/OLR1/FCAR/QPCT/CEACAM1/CXCL1/PGLYRP1/CD177/MMP9/PPBP/CRISP3/OLFM4/CAMP/LTF/HBB | 30 |
| CC | GO:0031838 | haptoglobin-hemoglobin complex | 8/1016 | 11/19869 | 6.54E-09 | 4.51E-07 | 3.90E-07 | HBQ1/HBG1/HBM/HBD/HBG2/HBA1/HBA2/HBB | 8 |
| CC | GO:0045121 | membrane raft | 42/1016 | 326/19869 | 3.49E-08 | 2.10E-06 | 1.82E-06 | S100A10/KCNE3/SELPLG/AHNAK/ADCY2/ERLIN1/MS4A4A/BTK/TGFBR2/FYN/RGMB/ADTRP/TNF/F2R/STOM/LRP6/ICAM1/MME/CDH1/IL6ST/DLL1/CARD11/BAALC/SLC2A1/DPP4/OLR1/CD48/SKAP1/ABCA1/KCNA3/ADD2/CD177/PTGS2/CD2/CD226/PTCH1/MAL/LCK/S1PR1/ZAP70/CD8A/APP | 42 |
| CC | GO:0098857 | membrane microdomain | 42/1016 | 327/19869 | 3.81E-08 | 2.10E-06 | 1.82E-06 | S100A10/KCNE3/SELPLG/AHNAK/ADCY2/ERLIN1/MS4A4A/BTK/TGFBR2/FYN/RGMB/ADTRP/TNF/F2R/STOM/LRP6/ICAM1/MME/CDH1/IL6ST/DLL1/CARD11/BAALC/SLC2A1/DPP4/OLR1/CD48/SKAP1/ABCA1/KCNA3/ADD2/CD177/PTGS2/CD2/CD226/PTCH1/MAL/LCK/S1PR1/ZAP70/CD8A/APP | 42 |
| MF | GO:0003823 | antigen binding | 51/1002 | 171/18432 | 2.12E-24 | 2.00E-21 | 1.80E-21 | DHCR24/LILRA1/HLA-B/IGHV3-30/IGHV2-5/IGHV1-69/IGHV3-74/IGHV3-48/IGHV4-39/IGLV3-25/IGLV1-47/IGLV3-19/IGLC7/IGHV3-33/IGLV6-57/IGKV3-15/IGHD/SLC7A5/IGHV5-51/IGLV3-1/IGLV1-44/TRDC/IGLV2-11/HLA-F/IGHM/IGLV2-23/IGHV3-15/IGLV3-21/LAG3/SLAMF1/IGLV2-8/IGLV1-40/IGHV3-21/CD48/IGHV3-7/IGKV3-20/IGKV1-5/KLRD1/IGLC2/IGHV3-23/IGLV2-14/IGKV4-1/IGHG4/IGHA2/IGLC3/IGHG3/IGHG2/TRBC2/IGHA1/IGHG1/IL7R | 51 |
| MF | GO:0034987 | immunoglobulin receptor binding | 26/1002 | 79/18432 | 3.11E-14 | 1.47E-11 | 1.32E-11 | IGHV3-30/IGHV2-5/IGHV1-69/IGHV3-74/IGHV3-48/IGHV4-39/IGLC7/IGHV3-33/IGHD/CLEC4D/IGHV5-51/TRDC/IGHM/IGHV3-15/IGHV3-21/IGHV3-7/IGLC2/IGHV3-23/IGHG4/IGHA2/IGLC3/IGHG3/IGHG2/TRBC2/IGHA1/IGHG1 | 26 |
| MF | GO:0031720 | haptoglobin binding | 8/1002 | 10/18432 | 3.03E-09 | 9.53E-07 | 8.56E-07 | HBQ1/HBG1/HBM/HBD/HBG2/HBA1/HBA2/HBB | 8 |
| MF | GO:0005344 | oxygen carrier activity | 9/1002 | 14/18432 | 6.26E-09 | 1.48E-06 | 1.33E-06 | HBQ1/IPCEF1/HBG1/HBM/HBD/HBG2/HBA1/HBA2/HBB | 9 |
| MF | GO:0019838 | growth factor binding | 24/1002 | 132/18432 | 1.65E-07 | 3.12E-05 | 2.80E-05 | FLT3/PDGFA/CHRDL1/TGFBR2/FGFR1/LTBP3/LTBP2/PCSK6/PDGFRB/A2M/IGFBP4/IL6ST/IL10RA/FLT4/COL1A1/IGFBP3/IL1RN/IL1R1/LRRC32/ITGB3/THBS1/FGFBP2/TGFBR3/IL2RB | 24 |
| MF | GO:0019955 | cytokine binding | 25/1002 | 144/18432 | 2.32E-07 | 3.65E-05 | 3.28E-05 | CSF1R/IL3RA/CSF2RA/CHRDL1/NBL1/TGFBR2/LTBP3/TNFRSF14/CXCR1/A2M/IL6ST/CXCR5/CXCR3/IL10RA/IL1RN/IL1R1/LRRC32/CCR4/GBP1/IL18R1/ITGB3/THBS1/CCR7/TGFBR3/IL2RB | 25 |
| MF | GO:0016684 | oxidoreductase activity, acting on peroxide as acceptor | 14/1002 | 57/18432 | 1.54E-06 | 0.000207857 | 0.00018658 | CAT/MGST2/HBQ1/GPX3/IPCEF1/SESN3/HBG1/PTGS2/HBM/HBD/HBG2/HBA1/HBA2/HBB | 14 |
| MF | GO:0005161 | platelet-derived growth factor receptor binding | 7/1002 | 14/18432 | 3.37E-06 | 0.000397241 | 0.000356578 | PDGFD/PDGFA/PTPRJ/PDGFRB/IL1R1/VEGFA/ITGB3 | 7 |
| MF | GO:0004896 | cytokine receptor activity | 18/1002 | 97/18432 | 4.20E-06 | 0.000440709 | 0.000395597 | FLT3/IL3RA/CSF2RA/CSF2RB/CXCR1/IL6ST/CXCR5/CXCR3/EPOR/IL10RA/IL21R/IL1R1/CCR4/IL18R1/CCR7/IL18RAP/IL7R/IL2RB | 18 |
| MF | GO:0140375 | immune receptor activity | 23/1002 | 148/18432 | 5.00E-06 | 0.000471908 | 0.000423601 | FLT3/LILRA1/IL3RA/CSF2RA/FCGR2B/CD160/CSF2RB/CXCR1/IL6ST/CXCR5/CXCR3/EPOR/IL10RA/KLRF1/IL21R/IL1R1/KLRD1/CCR4/IL18R1/CCR7/IL18RAP/IL7R/IL2RB | 23 |

**
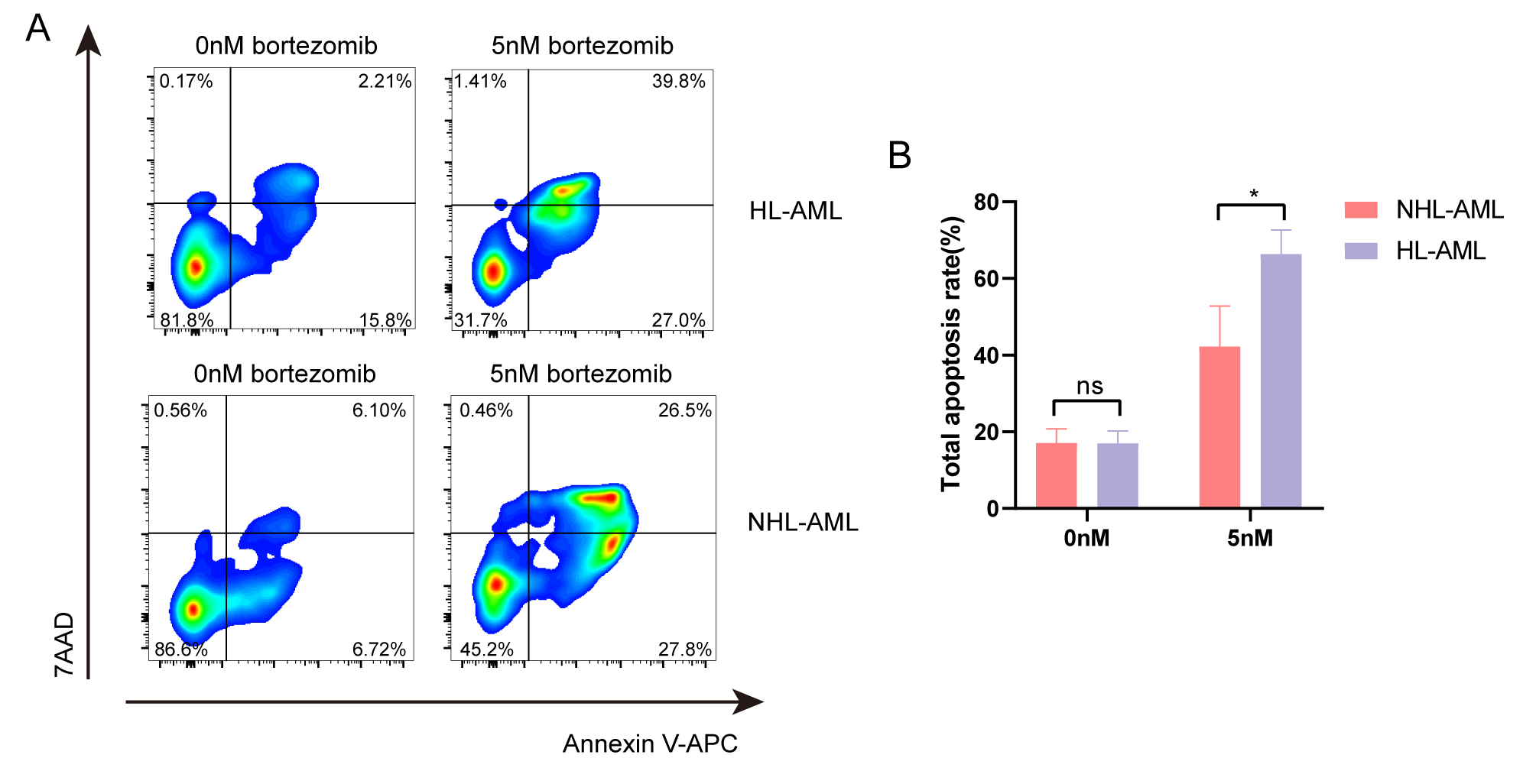
**

**Figure S1 Cytotoxic effects of bortezomib on HL-AML and NHL-AML cells**

Apoptosis rate detected by flow cytometry 48 hours post-bortezomib treatment (A) representative flow cytometry apoptosis plot; (B) apoptosis rate statistics.
